# Pathways and gene networks mediating the regulatory effects of cannabidiol, a nonpsychoactive cannabinoid, in autoimmune T cells

**DOI:** 10.1186/s12974-016-0603-x

**Published:** 2016-06-03

**Authors:** Ewa Kozela, Ana Juknat, Fuying Gao, Nathali Kaushansky, Giovanni Coppola, Zvi Vogel

**Affiliations:** The Dr Miriam and Sheldon G. Adelson Center for the Biology of Addictive Diseases, Sackler Faculty of Medicine, Tel Aviv University, Tel Aviv, 6997801 Israel; Department of Neurobiology, Weizmann Institute of Science, Rehovot, 76100 Israel; Departments of Psychiatry and Neurology, Semel Institute for Neuroscience and Human Behavior, David Geffen School of Medicine, University of California, Los Angeles, CA 90095 USA

**Keywords:** Autoimmune, Memory T cells, Gene expression, Cannabidiol

## Abstract

**Background:**

Our previous studies showed that the non-psychoactive cannabinoid, cannabidiol (CBD), ameliorates the clinical symptoms in mouse myelin oligodendrocyte glycoprotein (MOG)35-55-induced experimental autoimmune encephalomyelitis model of multiple sclerosis (MS) as well as decreases the memory MOG35-55-specific T cell (T_MOG_) proliferation and cytokine secretion including IL-17, a key autoimmune factor. The mechanisms of these activities are currently poorly understood.

**Methods:**

Herein, using microarray-based gene expression profiling, we describe gene networks and intracellular pathways involved in CBD-induced suppression of these activated memory T_MOG_ cells. Encephalitogenic T_MOG_ cells were stimulated with MOG35-55 in the presence of spleen-derived antigen presenting cells (APC) with or without CBD. mRNA of purified T_MOG_ was then subjected to *Illumina* microarray analysis followed by ingenuity pathway analysis (IPA), weighted gene co-expression network analysis (WGCNA) and gene ontology (GO) elucidation of gene interactions. Results were validated using qPCR and ELISA assays.

**Results:**

Gene profiling showed that the CBD treatment suppresses the transcription of a large number of proinflammatory genes in activated T_MOG_. These include cytokines (*Xcl1, Il3*, *Il12a, Il1b*), cytokine receptors (*Cxcr1*, *Ifngr1*), transcription factors (*Ier3, Atf3, Nr4a3, Crem*), and TNF superfamily signaling molecules (*Tnfsf11*, *Tnfsf14*, *Tnfrsf9*, *Tnfrsf18*). “*IL-17 differentiation*” and “*IL-6 and IL-10-signaling*” were identified among the top processes affected by CBD. CBD increases a number of IFN-dependent transcripts (*Rgs16, Mx2*, *Rsad2*, *Irf4*, *Ifit2*, *Ephx1*, *Ets2*) known to execute anti-proliferative activities in T cells. Interestingly, certain MOG35-55 up-regulated transcripts were maintained at high levels in the presence of CBD, including transcription factors (*Egr2*, *Egr1*, *Tbx21*), cytokines (*Csf2*, *Tnf*, *Ifng*), and chemokines (*Ccl3*, *Ccl4*, *Cxcl10*) suggesting that CBD may promote exhaustion of memory T_MOG_ cells. In addition, CBD enhanced the transcription of T cell co-inhibitory molecules (*Btla*, *Lag3*, *Trat1*, and *CD69*) known to interfere with T/APC interactions. Furthermore, CBD enhanced the transcription of oxidative stress modulators with potent anti-inflammatory activity that are controlled by Nfe2l2/Nrf2 (*Mt1*, *Mt2a*, *Slc30a1*, *Hmox1*).

**Conclusions:**

Microarray-based gene expression profiling demonstrated that CBD exerts its immunoregulatory effects in activated memory T_MOG_ cells *via* (a) suppressing proinflammatory Th17-related transcription, (b) by promoting T cell exhaustion/tolerance, (c) enhancing IFN-dependent anti-proliferative program, (d) hampering antigen presentation, and (d) inducing antioxidant milieu resolving inflammation. These findings put forward mechanism by which CBD exerts its anti-inflammatory effects as well as explain the beneficial role of CBD in pathological memory T cells and in autoimmune diseases.

**Electronic supplementary material:**

The online version of this article (doi:10.1186/s12974-016-0603-x) contains supplementary material, which is available to authorized users.

## Background

Natural (*Cannabis* derived), synthetic, and endogenous cannabinoids were shown to exert potent anti-inflammatory effects in various models of inflammation (reviewed by [1, 2]), including T cell-mediated autoimmunity [3]. However, most of the experiments focused on the effects of THC, the main psychoactive *Cannabis* constituent, and on THC-like ligands that interact with either the CB1 cannabinoid receptors (mostly expressed on neurons) or the CB2 receptors (abundant on immune cells). Another phytocannabinoid, cannabidiol (CBD) has been recently gaining a major interest as a potent immunomodulatory compound [4]. CBD has a very weak affinity toward the CB1 and CB2 cannabinoid receptors and thus lacks CB1-mediated psychoactivity [5]. Moreover, CBD proved to have very low toxicity when examined in humans [6].

Indeed, CBD was observed to induce anti-inflammatory effects in animal models of T cell-mediated collagen-induced arthritis [7], autoimmune diabetes [8], and autoimmune hepatitis [9]. Recently, we have reported that CBD administered systemically ameliorated clinical symptoms in mouse myelin oligodendrocyte glycoprotein (MOG)35-55-induced experimental autoimmune encephalitis (EAE) model of multiple sclerosis (MS), a neurodegenerative autoimmune disease resulting in progressing paralysis and initiated by autoreactive T cells targeting myelin sheaths [10, 11]. We showed that CBD diminishes CNS immune infiltration, microglial activation, and axonal damage in these EAE mice [12]. Our observations were confirmed by other groups [13–15]. The mechanisms of these beneficial regulatory CBD activities are not yet understood.

Autoimmune pathologies, including MS/EAE, are driven by transformed subsets of T cells called memory T cells. These autoreactive memory T cells are falsely primed by antigen-presenting cells (APC) to target own cells leading to tissue degeneration and disease development including type I diabetes, rheumatoid arthritis, and MS. Memory T cells exhibit high proliferation potential in response to self-antigens along with high pathogenic effector functions controlled by specific signaling pathways [16]. Autoimmune memory T cells (including those that target myelin sheath and lead to MS development) secrete interleukin (IL)-17 cytokine in retinoic acid receptor-related orphan receptor γ-T (RORγt)/signal transducer and activator of transcription 3 (STAT3)-dependent manner and were defined as autoimmune Th17 phenotype [17–19]. Adoptive transfer of such encephalitogenic T cell clones to healthy animals results in rapid and severe MS-like symptoms [20, 21] and antigen re-activation of quiescent, circulating memory T cells may contribute to MS relapses in relapsing–remitting MS forms [22]. Therapeutic targeting of these memory T cells seems to be difficult although this strategy proved to be efficient [23].

The effects of cannabinoids, including CBD, on these antigen-specific memory T cells driving autoimmune pathologies are not well described and the mechanisms of these activities are not known. We have recently shown that CBD is able to decrease the function of encephalitogenic Th17 cells. Using a highly myelin-specific memory T cell line recognizing the MOG35-55 myelin epitope (T_MOG_) we showed that CBD decreases the production and release of IL-17 from encephalitogenic T_MOG_ cells as well as of IL-6 [24], a cytokine controlling Th17 differentiation [25]. CBD also decreased the phosphorylation of STAT3 [26], a pathway known to control Th17-like function of memory T_MOG_ cells [27]. In parallel, we observed that CBD boosted anti-inflammatory processes in these activated memory T cells including increased production of anti-inflammatory IL-10 cytokine and increased activity of several regulatory transcription factors including STAT5 and EGR2 [26].

To study the transcriptional mechanisms involved in the CBD immunoregulatory effects, we profiled gene expression in total mRNA isolated from activated T_MOG_ cells treated with CBD using microarrays. Detailed bioinformatics analyses allowed us to identify gene networks, pathways and upstream regulators that mediate the CBD suppressory effects. We show that CBD downregulates the transcription of various proinflammatory genes controlling Th17 function of encephalitogenic T cells while enhancing IFN-dependent anti-proliferative genetic program and potentiating the expression of genes hampering APC/T communication and antigen presentation. Moreover, a number of anti-oxidant transcripts exerting anti-inflammatory effects were upregulated by CBD in activated memory T cells.

## Methods

### Reagents

Lyophilized MOG35-55 peptide [MEVGWYRSPFSRVVHLYRNGK] purchased from GenScript (Piscataway, NJ, USA) was reconstituted in sterile PBS and the stock solution stored in aliquots at −20 °C. CBD (kindly obtained from Prof. Raphael Mechoulam, the Hebrew University of Jerusalem, Israel) was dissolved in ethanol. The dose of 5 μM CBD used here was selected based on our previous studies in which we showed that CBD at this concentration significantly inhibits MOG35-55-induced T_MOG_ cell proliferation and their Th17 activity, i.e., IL-17 expression and release [12, 24, 26]. Fetal calf serum (FCS) and other tissue culture reagents were obtained from *Biological Industries* (Kibbutz Beit HaEmek, Israel).

### T_MOG_ stimulation and CD4^+^ microbead purification from APC/T_MOG_ co-cultures

The MOG35-55-specific T cell line (T_MOG_) was maintained as described before [12, 24, 26, 28]. APCs were freshly isolated from spleens of 8-week naïve male C57BL/6 mice just before the experiments. Dissociated spleen cells were plated (50 × 10^6^ cells/10 cm plate) in RPMI-1640 medium containing 2.5 % FCS, 100 μg/ml streptomycin, 100 U/ml penicillin, 2 mM L-glutamine, and 50 μM β-mercaptoethanol. After 2 h at 37 °C in 5 % CO2 humidified air to allow APC adherence, the media with non-adherent cells was removed and the adherent APCs gently washed with Ca^++^/Mg^++^ containing PBS and covered with a new medium. Then, 2.5 × 10^6^ of T_MOG_ cells were added and APC/T_MOG_ co-cultures were stimulated immediately with 5 μg/ml of MOG35-55 for 8 h in the presence or absence of CBD at 5 μM. The incubation time of 8 h was chosen based on previous time- and dose-response experiments [24, 26, 29]. CBD was added just prior to the addition of the MOG35-55. After 8 h of incubation, the media containing mostly T_MOG_ cells (but not the adherent APC cells) were carefully collected and spun down for 10 min at 2,000 rpm. The cell pellet was washed in PBS containing 0.5 % BSA and 2 mM EDTA, centrifuged again, and resuspended in 90 μl of this buffer. To improve the purity of collected cells, CD4 (L3T4) magnetic beads (*Miltenyi Biotec GmbH,* Bergisch Gladbach, Germany) were added to the cell suspension for positive selection of CD4^+^ cells as described earlier [26, 29]. The mRNA isolated from purified T_MOG_ cells was subjected to global mRNA microarray expression analysis followed by quantitative real time reverse transcription polymerase chain reaction (qPCR) for validation of selected gene products.

### RNA extraction, microarray transcript analysis, and validation by qPCR

Purified T_MOG_ cells were lysed and subjected to RNA extraction (*5Prime*, Darmstadt, Germany) as described earlier [26, 29, 30]. For comparative microarray analysis, 200 ng of total RNA were amplified, labeled and hybridized onto *Illumina MouseRef-8 v 2.0* Expression Bead-Chip (*Illumina Inc*., San Diego, CA, USA) as described earlier [26, 29]. Raw data were log2 transformed and normalized using quantile normalization. The data is presented throughout the manuscript as fold change unless stated otherwise. Statistical microarray analysis and gene expression analysis of the raw data were performed at the Informatics Center for Neurogenetics and Neurogenomics core at UCLA using R scripts (www.r-project.org) and Bioconductor packages (http://www.bioconductor.org; [31]) as described [32]*.*

Pathway and global functional analyses were performed using QIAGEN’s Ingenuity Pathway Analysis (IPA®, QIAGEN Redwood City CA, USA www.qiagen.com/ingenuity). Genes that met the *p* value cutoff of 0.005 for differential expression were used to build the gene networks using IPA tools.

Weighted Gene Co-expression Network Analysis (WGCNA; [33]) (http://labs.genetics.ucla.edu/horvath/htdocs/CoexpressionNetwork/) was applied to complete the functional characterization of gene expression data. WGCNA is an analysis method that recognizes co-expression networks based on topological overlap between genes and considers the correlation of two genes with each other and the degree of their shared correlations within the network. Briefly, genes consistently present on arrays with high coefficient of covariation and high connectivity were selected for network construction and were hierarchically clustered. Clusters of highly interconnected genes (modules) were determined based on their topological overlap using a dynamic tree-cutting algorithm. Such modules were visualized using VisANT (http://visant.bu.edu). The gene expression pattern was condensed within a module to a “module eigengene” (ME), which is a weighted summary of gene expression in the module and can be correlated to traits. The relationships between genes within modules can be identified, and follow up analyses can focus on hub genes (genes with highest connectivity in the module) and corresponding pathways driving gene expression changes without any a priori assumptions about gene function. At the final stage, the modules of interest were annotated using gene ontology (GO) functional and biological categories using online tool (http://geneontology.org). The WGCNA method has been used in a large number of recent transcriptional studies to reveal functional gene networks [34, 35].

### qPCR analysis

Selected gene products found by microarray analysis to be affected by CBD were validated by qPCR as described [26, 30]. The cDNA of each chosen gene was amplified using a pair of specific primers presented in Additional file 1: Table S1. β2-microglobulin (β2mg) gene product was used for normalization [30]. The qPCR runs were repeated 3–4 times using mRNA preparations from independent experiments.

### ELISA assay

The enzyme-linked immunosorbent assay (ELISA) was performed as described earlier [24]. Shortly, the T_MOG_ cells were cultured in assay medium in 24-well plates (0.25 × 10^6^ cells per well) together with splenic APC (5 × 10^6^ cells/well). CBD at 5 μM was added to the cells just before the addition of MOG35-55 at 5 μg/ml. After 24 h of incubation, the cell-conditioned media were collected, spun down, and analyzed for IL-1β and IL-3 concentrations by ELISA (*R&D Systems*, Minneapolis, MN, USA). The incubation time of 24 h was chosen based on our previous observations [24].

### Statistical analysis of qPCR and ELISA data

qPCR and ELISA data are expressed as the mean ± SEM of 3–4 independent experiments and analyzed for statistical significance using one way analysis of variance (ANOVA), followed by Newman-Keul’s post-hoc test. *p* < 0.05 was considered significant. *Graph Pad Prism* program (La Jolla, CA, USA) was used for statistical analysis of the data.

## Results

### Gene expression profile of activated T_MOG_ cells treated with CBD

We recently described the detailed gene expression profile of T_MOG_ activated by MOG35-55 [29] and demonstrated a powerful proinflammatory effect of MOG35-55 activation at the transcriptional level confirming the Th17 function of activated T_MOG_ cells [24, 26].

Here, we examined the effects of CBD co-incubation on MOG35-55-induced transcriptional profile of T_MOG_. Samples of mRNA were prepared from purified T_MOG_ cells (CD4^+^ positive selection), co-cultured earlier with pre-attached APC, and stimulated with MOG35-55 at 5 μg/ml for 8 h in the presence or absence of CBD at 5 μM.

**Fig. 1:**
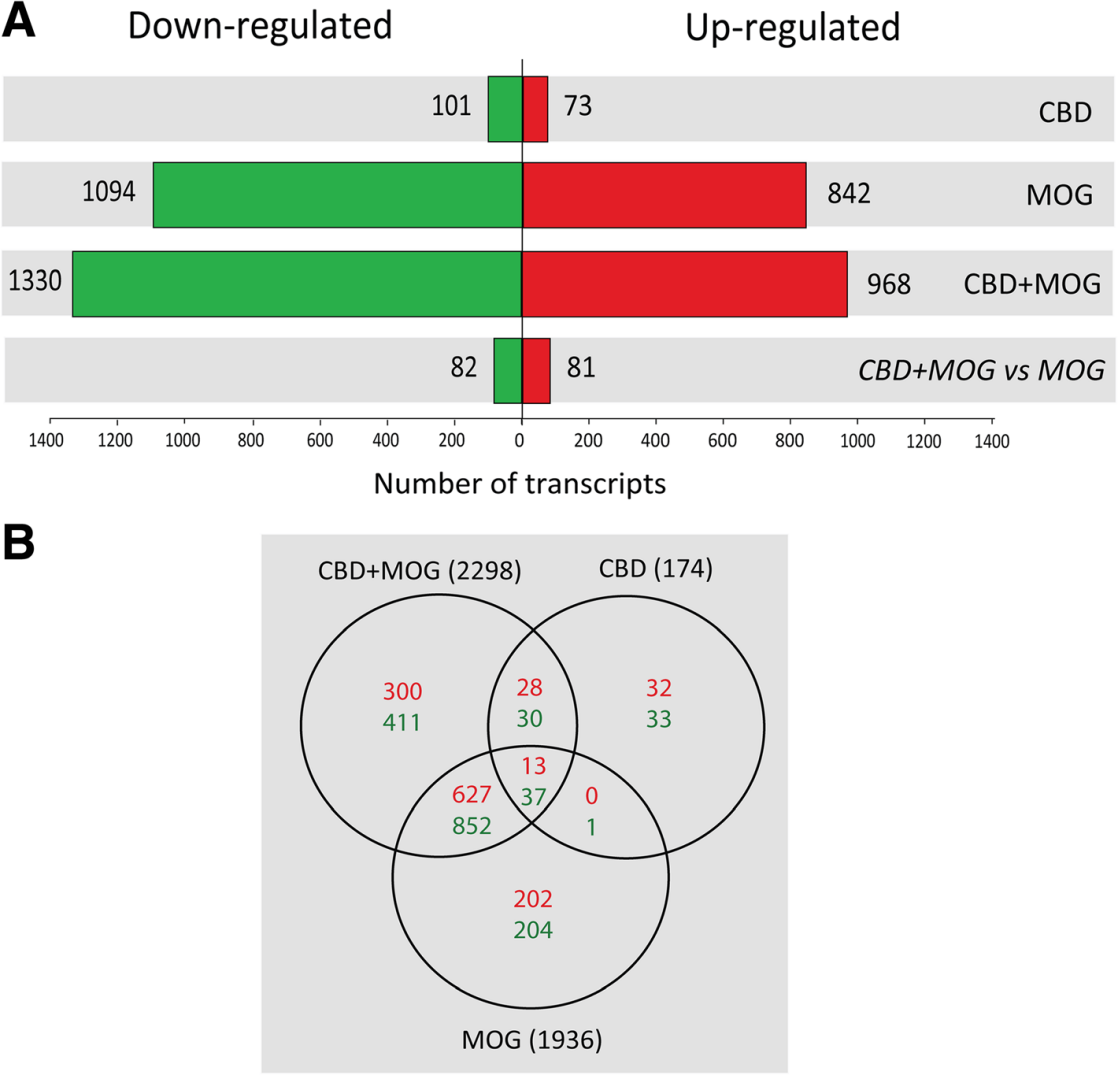
Number of gene transcripts affected in MOG35-55-stimulated T_MOG_ cells in the presence or absence of CBD. mRNA prepared from T_MOG_ cells co-cultured with APC and stimulated with MOG35-55 in the presence or absence of CBD was subjected to microarray analysis as described in Methods. **a** Number of differentially expressed transcripts that were either significantly upregulated (*red*) or downregulated (*green*) across the different treatment conditions *vs* control untreated cells (*p* < 0.005). **b** Venn’s diagram illustrating the number of transcripts affected (up- and downregulation) by CBD, MOG35-55, or CBD+MOG35-55 treatments (*p* < 0.005) and their overlap

Microarray analysis based on the threshold of *p* < 0.005 revealed that 2755 transcripts (within ~25,600 targets present on this *Illumina* set) were differentially regulated across the treatments. Of these, stimulation with MOG35-55 upregulated the expression of 842 gene transcripts and downregulated 1094 gene probes (Fig. 1a). Addition of CBD to MOG35-55-stimulated T_MOG_ cells resulted in a total of 968 upregulated gene transcripts and in 1330 downregulated transcripts as compared to control (without CBD and MOG35-55) samples. Out of these, 81 gene products were significantly (*p* < 0.005) upregulated by the addition of CBD and 82 gene products were downregulated by CBD as compared to the level observed in MOG35-55-stimulated cells without CBD (Fig. 1b). T_MOG_ cells stimulated with MOG35-55 alone or co-incubated with CBD shared the same 627 upregulated and 852 downregulated gene products. Twenty eight transcripts were upregulated and 30 downregulated in T_MOG_ cells incubated with CBD given alone or by CBD+MOG35-55 combination. Only one gene product was downregulated by MOG35-55-only stimulation and by CBD-only treatment. Interestingly, 13 individual transcripts were always upregulated and 37 were always downregulated by all experimental conditions.

### Gene by gene analysis

Only genes reaching the criteria of *p* < 0.005 and fold change of 0.2 (i.e., 20 % change) were included in further analysis of CBD effects on transcriptional activity of stimulated T_MOG_. Several approaches were employed to characterize the CBD modulation of self-antigen activation of T_MOG_. These included (1) identification of the most CBD-suppressed transcripts within those activated by MOG35-55, (2) identification of MOG35-55-upregulated transcripts that were not affected by CBD, (3) identification of the most CBD-upregulated transcripts, and (4) identification of the effect of CBD on the MOG35-55 downregulated genes.

Table 1 displays the transcripts upregulated by stimulation with MOG35-55 and significantly reduced in the presence of CBD. These transcripts include: cytokines such as *Il3* mRNA (downregulated by CBD by 72 %), *Il1b* mRNA (reduced by 70 %), *Xcl1* (by 49 %), and *Il12a* (by 24 %); cytokine and chemokine receptors’ mRNAs (e.g., *Ifngr1* by 60 %, *Cxcr1* by 50 %); transcription factors and regulators (*Crem* by 50 %, *Ier3* by 31 %, *Atf3* by 28 %); TNF-family signaling members (*Tnfrsf18* by *40 %, Tnfsf11* and *Tnfsf14* by 30 %, *Tnfrsf9* by 21 %); signaling elements including those affecting adhesion (*Amica1* by 21 %), growth (*Igfbp4* by 50 %), cell structure and trafficking (*Vps37b* by 60 %, *Tubb2d* by 50 %) as well as lipid/cholesterol synthesis and metabolism (*Dgat1* by 60 %, *Fdps* by 50 %). These changes indicate that CBD has a profound suppressing effect on various MOG35-55-activated genes in T_MOG_ cells. Several of the gene transcripts that were reduced by the combined CBD+MOG35-55 treatment (*vs* MOG35-55 only) were also reduced by incubation with CBD alone (*vs* control) including *Il1b* (by 70 %), *Cxcr1* (by 30 %), *Ifngr1* (by 60 %), *Crem* (by 50 %), *Tnfrs18* (by 50 %), *Igfbp4* (by 50 %), *Vps37b* (by 60 %), *Tubb2b* (by 50 %), *Fdps* (by 50 %), and *Dgat1* (by 60 %).

**Table 1:** List of transcripts which expression is upregulated by MOG35-55 stimulation and this upregulation is suppressed by CBD (*p* < 0.005)

Symbol	Gene name	Accession number	MOG alone [fold]^$^	MOG+CBD [fold]^$^	Change [%]	CBD alone [fold]^$^
Cytokines, chemokines, and receptors						
Xcl1	Chemokine (C motif) ligand 1	NM_008510.1	30.0	15.3	−49 %	1.0
Il3	Interleukin 3	NM_010556.2	25.4	7.2	−72 %	1.0
Cxcl9	Chemokine (C-X-C motif) ligand 9	NM_008599	7.4	5.4	−27 %	0.9
Gpr83	G protein-coupled receptor 83	NM_010287	3.9	1.8	−53 %	0.9
Il12a	Interleukin 12A	NM_008351	2.3	1.5	−24 %	0.9
Cxcr1	Chemokine (C-X-C motif) receptor 1 (Il8ra, CD181)	NM_178241.1	1.7	0.9	−50 %	*0.7*
Il1b	Interleukin 1, beta	NM_008361	1.5	0.5	−70 %	*0.3*
Ifngr1	Interferon gamma receptor 1	NM_10511.1	0.8	0.3	−60 %	*0.4*
Transcription factors and regulators						
Ier3	Immediate early response 3	NM_133662.1	5.5	3.8	−31 %	1.3
Atf3	Activating transcription factor 3	NM_007498.2	4.7	3.4	−28 %	1.1
Nr4a3	Nuclear receptor subfamily 4, group A, member 3	NM_015743.1	4.1	3.2	−22 %	1.0
Crem	cAMP responsive element modulator	NM_013498	1.9	0.9	−50 %	*0.5*
TNF-family signaling elements						
Tnfsf11	Tumor necrosis factor ligand superfamily member 11 (*Receptor activator of nuclear factor kappa-B ligand (RANKL))*	NM_011613.2	11.8	8.2	−31 %	0.9
Tnfsf14	Tumor necrosis factor ligand superfamily member 14	NM_019418.1	5.0	3.5	−30 %	1.0
Tnfrsf9	Tumor necrosis factor receptor superfamily, member 9	NM_011612.2	4.2	3.3	−21 %	0.8
Tnfrsf18	Tumor necrosis factor receptor superfamily, member 18	NM_009400.1	1.3	0.7	−40 %	*0.5*
Signaling elements						
Sphk1	Sphingosine kinase	NM_011451	12.3	8.2	−34 %	1.0
Amica1	Adhesion molecule interacting with CXADR antigen	NM_001005421.2	5.0	3.9	−21 %	1.2
Axud1	Cysteine/serine-rich nuclear protein 1/TGF-beta induced apoptosis protein 3	NM_153287.2	4.4	3.5	−20 %	0.9
Cdk5r1	Cyclin-dependent kinase 5 activator 1	NM_009871.2	3.9	3.1	−20 %	0.9
Igfbp4	Insulin-like growth factor binding protein 4	NM_010517.2	2.3	1.1	−50 %	*0.5*
Vps37b	Vacuolar protein sorting 37 homologue B	NM_177876.2	1.5	0.6	−60 %	*0.4*
Dgat1	Diacylglycerol O-acyltransferase 1	NM_010046.2	1.0	0.4	−60 %	*0.4*
Tubb2d	Tubulin, beta 2b	NM_023716.1	0.8	0.5	−50 %	*0.5*
Fdps	Farnesyl diphosphate synthase	NM_134469	0.8	0.4	−50 %	*0.5*

^$^The results are presented as fold change *vs* control (without MOG35-55 or CBD). Numbers in italics indicate a significant effect of CBD alone (p < 0.005) in non-stimulated T_MOG_

It is important to note (see Table 2) that several of the MOG35-55-upregulated proinflammatory transcripts were not affected by CBD (given in the presence of MOG35-55). These include transcripts of several cytokines (e.g., *Csf2*, *Tnf, Ifng*), MOG35-55-upregulated chemokines (e.g., *Ccl3*, *Ccl4*, *Cxcl10*), regulatory glycoproteins (e.g., *Sema7a*), transcription factors mediating tolerogenic mechanisms (*Egr2*, *Egr1*), and several IFN-related transcriptional and signaling regulators (e.g., *Tbx21*, *Ifit3*).

**Table 2:** List of transcripts which expression is upregulated by MOG35-55 stimulation and not affected by CBD

Symbol	Gene name	Accession number	MOG alone [fold]^$^	MOG+CBD [fold]^$^	CBD alone [fold]^$^
Cytokines					
Csf2	Colony stimulating factor 2	NM_009969.2	77.0	67.1	1.1
Tnf	Tumor necrosis factor alpha	NM_013693.1	15.3	13.8	1.1
Ifng	Interferon gamma	NM_008337.1	10.8	9.5	0.8
Chemokines					
Ccl3	Chemokine (C-C motif) ligand 3	NM_011337.1	54.1	48.6	0.9
Ccl4	Chemokine (C-C motif) ligand 4	NM_013652.1	40.2	37.3	0.9
Cxcl10	Chemokine (C-X-C motif) ligand 10	NM_021274.1	9.0	9.8	0.9
Glycoproteins					
Sema7a	Semaphorin 7A (GPI membrane anchor)	NM_011352.2	4.6	4.3	0.8
Transcription factors					
Egr2	Early growth response 2	NM_010118.1	20.1	18.9	*1.3*
Egr1	Early growth response 1	NM_007913.2	10.7	11.7	*1.5*
Tbx21	T-box 21	NM_019507.1	2.2	2.5	*0.9*
*Signaling elements*					
Ndrg1	N-myc downstream regulated 1	NM_010884.1	6.2	6.7	*2.5*
Ifit3	Interferon-induced protein with tetratricopeptide repeats 3	NM_010501.1	5.6	5.9	0.9

^$^The results are presented as fold change *vs* control (without MOG35-55 or CBD). Numbers in italics indicate a significant effect of CBD alone (*p* < 0.005)

Gene transcripts that were significantly upregulated in the presence of CBD in MOG35-55-stimulated T_MOG_ cells are listed in Table 3. Among these transcripts we find mainly membrane and transmembrane molecules known to negatively regulate T cell activation, e.g., *via* disruption of APC/T interactions as well as of antigen processing and presentation. These include transcripts of *Lag3* (increase of 210 %), *Btla* (80 %), and *CD69* (39 %). Moreover, CBD given alone or in combination with MOG35-55 enhanced the transcription of *Trat1* (an intracellular element trafficking CTLA-4 inhibitory T cell receptor into the membrane) by 60 and 200 %, respectively. CBD had a remarkable boosting effect on the transcriptional activity of numerous IFN-dependent genes known to execute IFN-driven anti-proliferative responses including *Mx2*, *Irf4*, *Ifit2*, *Ephx1*, *Rsad2* (*Viperin*), *Ets2*, *Trim30*, and *Tyki* mRNAs. In addition, CBD had an enhancing effect on a number of transcripts associated with GTP activation and signaling, protein transport, and turnover in T cells such as *Tagap*, *Gfod1*, *Tnfsf8* (*CD30*), *Nfatc1*, *Gbp11*, *Ppic*, *Dusp6*, and *Slc3a2* (*CD98*) mRNAs.

**Table 3:** List of genes which transcription is enhanced by CBD in T_MOG_ cells (*p* < 0.005)

Symbol	Gene name	Accession number	MOG alone [fold]^$^	MOG+CBD [fold]^$^	Change [%]	CBD alone [fold]^$^
MOG35-55-upregulated and CBD-enhanced						
Surface negative regulators						
Lag3^&^	Lymphocyte-activation gene 3	NM_008479.1	1.7	5.2	+210 %	*1.7*
Btla	B and T lymphocyte associated	NM_177584.3	1.8	3.2	+80 %	*2*
Cd69	Cluster of Differentiation 69	XM_132882.1	3.9	5.4	+39 %	*1.4*
IFN-dependent transcripts						
Rsad2	Radical S-adenosyl methionine domain containing 2	NM_021384.2	4.9	9.5	+94 %	*1.6*
Trim30	Tripartite motif-containing 30	NM_009099	2.2	3.1	+40 %	1.1
Mx2	Myxovirus (influenza virus) resistance 2	NM_013606	6.0	8.2	+37 %	1.0
Irf4	Interferon regulatory factor 4	NM_013674.1	4.5	5.3	+18 %	*1.4*
Signaling elements						
Gbp11	Guanylate binding protein 11	NM_01039647.1	3.2	7	+120 %	1.1
Ppic	Peptidylprolyl isomerase C	NM_008908.1	1.8	3.2	+80 %	*1.8*
Tyki	Thymidylate kinase family LPS-inducible member	NM_020557.3	4	5.7	+42 %	1
Tagap	T-cell activation RhoGTPase activating protein	NM_145968.1	4.7	6.4	+35 %	*1.4*
Tnfsf8	Tumor necrosis factor (ligand) superfamily, member 8	NM_009403.2	2.2	2.9	+31 %	1.1
Dusp6	Dual specificity phosphatase 6	NM_026268.1	3.4	4.3	+26 %	*1.4*
Gfod1	Glucose-fructose oxidoreductase domain containing 1	NM_001033399.1	6.2	7.8	+26 %	1.0
Nfatc1^&^	Nuclear factor of activated T-cells, cytoplasmic 1	NM_16791	3.3	4	+21 %	*1.3*
Only CBD upregulated						
Anti-oxidant and anti-inflammatory						
Mt1	Metallothionein 1	NM_013602.2	0.5	2.3	+330 %	*3*
Slc30a1	Solute carrier family 30 (zinc transporter), member 1	NM_009579	0.9	1.8	+100 %	*1.8*
Mt2	Metallothionein 2	NM_008630.1	1	1.9	+80 %	*2.3*
Negative regulators of T cell activity						
Trat1	T cell receptor associated transmembrane adaptor 1	NM_198297	0.7	2.1	+200 %	*1.6*
IFN-dependent transcripts						
Ephx1	Epoxide hydrolase 1	NM_010145.2	0.7	1.8	+170 %	*2.4*
Ets2	V-ets avian erythroblastosis virus	NM_011809.2	1.3	2.4	+80 %	*1.3*
Signaling elements						
Ddr1	Discoidin domain receptor tyrosine kinase 1	NM_007584.1	1.1	3	+170 %	*2.4*
Pitpnm2	Phosphatidylinositol transfer protein, membrane-associated 2	NM_011256.1	1.2	2.9	+150 %	*2.6*
Gpnmb	Glycoprotein (transmembrane) nmb	NM_053110.2	0.4	0.9	+90 %	*2.2*
Asb2	Ankyrin repeat and SOCS box-containing protein 2	NM_023049.1	0.29	0.52	+79 %	*1.38*
Slc3a2	Solute carrier family 3 member 2	NM_008577.2	1.3	2.3	+70 %	*1.5*

^$^The results are presented as fold change *vs* control (without MOG35-55 or CBD). Numbers in italics indicate a significant effect of CBD (*p* < 0.005) in non-stimulated T_MOG_. ^&^result reported previously [25]

**Fig. 2:**
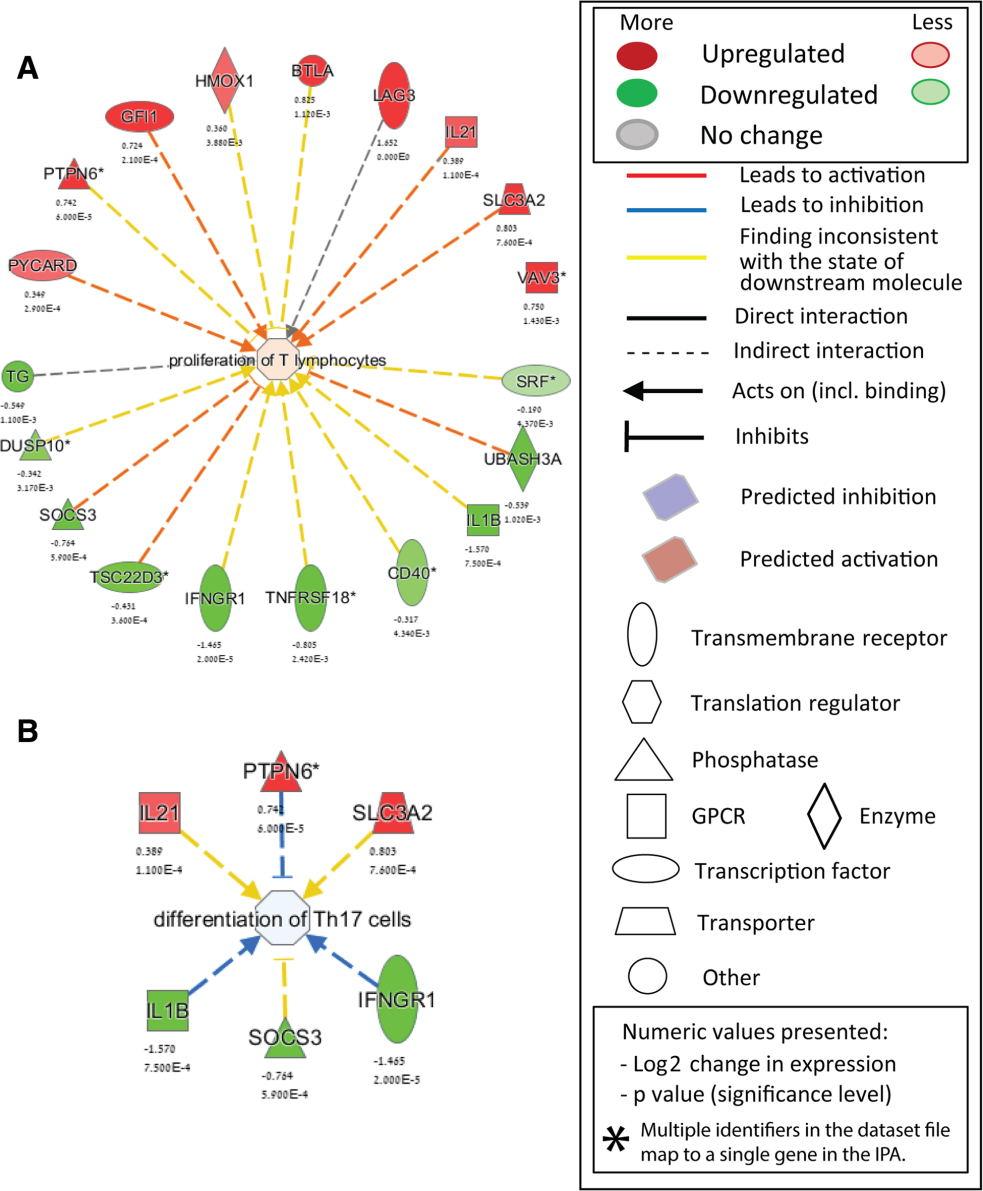
IPA-identified CBD-enriched functional terms in activated T_MOG_ cells. **a** Genes forming “*Proliferation of T lymphocytes*” annotation found to be affected by CBD (**b**). Genes forming “*Differentiation of Th17 cells*” annotation found to be affected by CBD. The direction of change is color-coded with *green* implying downregulation and *red* implying upregulation. Log2 values expressing CBD-induced change *vs* MOG35-55-only-stimulated cells and respective *p* values are provided below each transcript name. Explanation of symbols is provided in the *Legend*

**Fig. 3:**
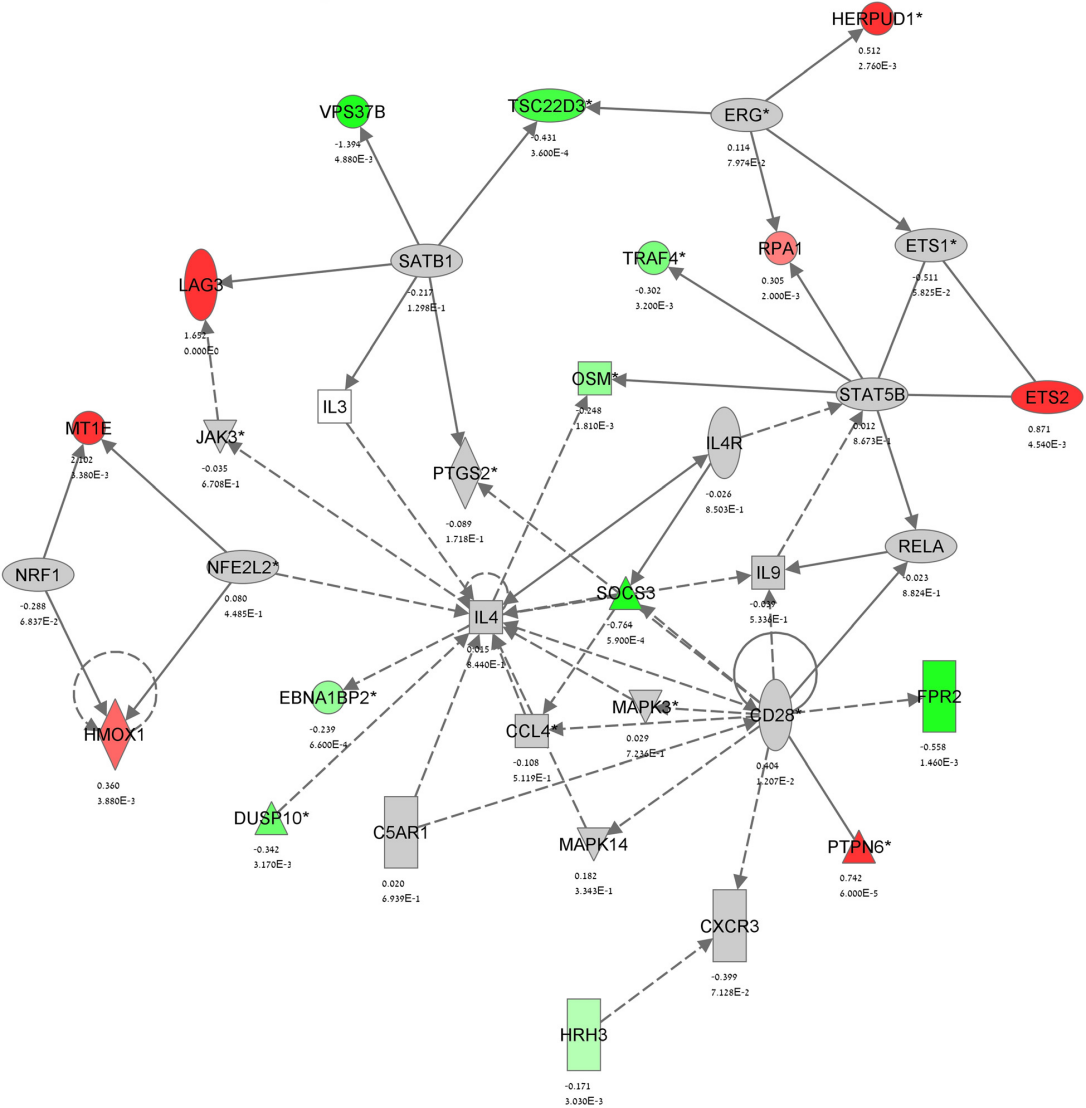
IPA gene network analysis of CBD effect in stimulated T_MOG_ cells—Network 1. Network analysis of the genes which expression is significantly (*p* < 0.005) affected by CBD in MOG35-55-stimulated T_MOG_ cells was performed using the IPA software as described in Methods. The network display, symbols, and biological relationships between the genes are explained in Fig. 2. Genes in *grey* were shown in the literature to interact with the colored gene products that appear in the scheme

Several transcripts including anti-oxidant and anti-inflammatory as well as negative regulators of T cell activity were increased by CBD even that they were not affected by MOG35-55. These included the zinc transporter *Slc30a1*, metallothioneins (*Mt1* and *Mt2*), heme oxygenase (*Hmox1*), and mRNA of a protein preserving endoplasmic reticulum homeostasis (*Herpud1*) (Table 3, Figs. 2 and 3).

At the last step, we focused on MOG35-55-downregulated transcripts and studied the effect of CBD on their levels. Table 4 shows a list of several highly MOG35-55-suppressed transcripts that were not affected by CBD. The list comprises of representatives of various gene families and functions. Interestingly, MOG35-55-activation led to a dramatic decrease in transcription of several IL-17 receptors (*Il17re*, *transcript variant 2*, *Il17re variant 1*, and *Il17rc*; see also [29]) and this downregulation was not affected by the presence of CBD.

**Table 4:** MOG35-55-most-downregulated genes (*p* < 0.005) that are not affected by CBD

Symbol	Definition	Accession	MOG alone [fold]^$^	MOG+CBD [fold]^$^	CBD alone [fold]^$^
Il17re	Interleukin 17 receptor E, variant 2	NM_001034029.1	0.23	0.23	1.04
Sytl2	Synaptotagmin-like 2, variant 5	NM_001040088.1	0.25	0.27	1.03
Il17re	Interleukin 17 receptor E, variant 1	NM_145826.2	0.26	0.27	1.11
Tmem176a	Transmembrane protein 176A	NM_025326.2	0.28	0.30	1.07
Ian3	Immune associated nucleotide 3	NM_146167.2	0.29	0.23	0.94
Klrd1	Killer cell lectin-like receptor, subfamily D, member1	NM_010654.1	0.30	0.29	0.95
Klri2	Killer cell lectin-like receptor family I member 2	NM_177155.2	0.31	0.35	1.01
Sit1	Suppression inducing transmembrane adaptor 1	NM_019436.1	0.32	0.29	0.84
Il17rc	Interleukin 17 receptor C	NM_134159.2	0.33	0.36	0.95
Golph2	Type II Golgi transmembrane protein	NM_027307	0.35	0.35	0.94

^$^The results are presented as fold change *vs* control (without MOG35-55 or CBD)

### Identification of functional networks affected by CBD in activated T_MOG_

As described above, CBD treatment of the MOG35-55 treated T_MOG_ cells resulted in upregulation of 81 transcripts and in downregulation of 82 transcripts. These genes were uploaded onto the IPA analysis program to identify the functional subsets, pathways, gene networks, and upstream regulators targeted by CBD. Figure 2 illustrates two IPA-identified CBD-enriched GO-functional terms in activated T_MOG_ cells, “*Proliferation of T lymphocytes*” (Fig. 2a) and “*Differentiation of Th17 cells*” (Fig. 2b). The “*Proliferation of T lymphocytes*” functional annotation was recognized by IPA to be upregulated in activated T_MOG_ as a result of CBD co-incubation due to upregulated transcription of *Pycard*, *Ptpn6*, *Gfi1*, *Hmox1*, *Btla*, *Lag3*, *Il21*, *Slc3a2* and *Vav3* mRNAs and due to parallel suppression of *Tg*, *Dusp10*, *Socs3*, *Tsc22d3*, *Ifngr1*, *Tnfsf18*, *Cd40*, *Il1b*, *Ubash3a* and *Srf* mRNAs (Fig. 2a). In addition, within the “*Differentiation of Th17 cells*" annotation, IPA classified the upregulated *Il21*, *Ptpn6*, *Slc3a2* and the downregulated *Il1b*, *Socs3*, and *Ifngr1* transcripts and recognized it to be suppressed by CBD in stimulated T_MOG_ (Fig. 2b).

IPA analysis identified several other canonical pathways that are affected by CBD in MOG35-55-stimulated T_MOG_ (Table 5). These included “*IL-10 signaling*” and “*IL-6 signaling*” (*p* < 0.005), followed by “*cAMP-mediated signaling*”, “*IGF-1 signaling*”, “*Fcg receptor mediated phagocytosis in macrophages and monocytes*”, “*Superpathway of cholesterol biosynthesis*”, and “*T helper cell differentiation*” pathways (p < 0.05).

**Table 5:** IPA-identified “*top canonical pathways*” enriched by CBD treatment in stimulated T_MOG_ cells

Canonical pathways	Annotated genes	*p* value
IL-10 signaling	*Hmox1*, *Il1b*, *Jun*, *Socs3*	0.0039
IL-6 signaling	*Il1b*, *Jun*, *Socs3*, *Srf*, *Vegfa*	0.0042
cAMP-mediated signaling	*Crem*, *Fpr2*, *Gnal*, *Grm6*, *Hrh3*	0.024
IGF-1 signaling	*Igfbp4*, *Jun*, *Socs3*, *Srf*	0.015
Fcγ receptor mediated phagocytosis in macrophages and monocytes	*Appc3*, *Hck*, *Hmox1*, *Vav3*	0.012
Superpathway of cholesterol biosynthesis	*Fdps*, *Sqle*	0.016
T helper cell differentiation	*CD40*, *Ifbgr1*, *Il21*	0.042

**Fig. 4:**
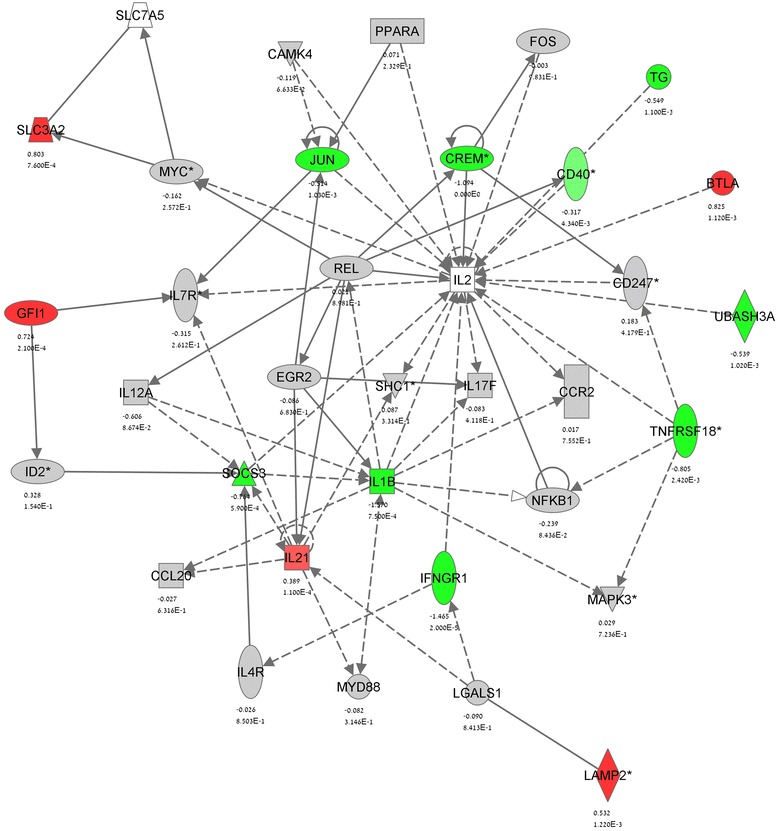
IPA gene network analysis of the CBD modulation of stimulated T_MOG_ cells—Network 2. See Figs. 2 and 3 for details

IPA identified the gene networks affected by CBD in MOG35-55 stimulated T_MOG_ cells (Figs. 3 and 4). Within these gene networks IPA denoted the main upstream regulators which function is modulated by CBD. Accordingly, *Network 1* (Fig. 3) links the effects of CBD to the modulation of IL-4, STAT5B, SATB1, CD28, NRF2/NFE2L2, and NRF1 while *Network 2* (Fig. 4) links the effects of CBD to IL-2 and IL-1β as well as to NFKB1 and EGR2.

Altogether, we show here that more cytokines, chemokines, receptors, and signaling pathways were down-regulated by CBD (Table 1, Figs. 1 and 2) than up-regulated (Table 3) which is in agreement with the reduction of T_MOG_ cell proliferation and their Th17 function shown by us before [12, 24]. However, among up-regulated genes we found mainly regulatory T cell molecules known to inhibit T cell proliferation and function.

### Validation by ELISA and qPCR

**Fig. 5:**
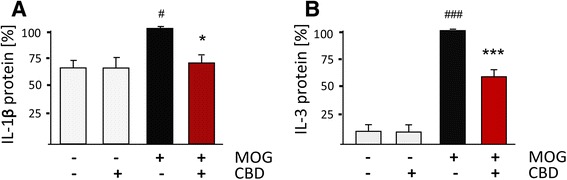
ELISA of IL-1β and IL-3 cytokines released from MOG35-55-stimulated T_MOG_ cells in the presence or absence of CBD. APC/T_MOG_ were stimulated with MOG35-55 at 5 μg/ml for 24 h in the presence or absence of CBD at 5 μM and the amounts of (**a**) IL-1β and (**b**) IL-3 in the culture media determined. *100 %* is equivalent to maximal MOG35-55 effect which refers to 15 ± 6 pg/ml for IL-1β and to 377 ± 100 pg/ml for IL-3. Statistics (one way ANOVA) and symbols: **a** F(3,6) = 6.7, *p* < 0.05. **b** F(3,15) = 80.25, *p* < 0.001; Symbols: ^#^ *p* < 0.05, ^###^ *p* < 0.01 *vs* Control, *p < 0.05, ***p < 0.001 *vs* MOG-35-55-stimulated cells

Several gene products that were identified by microarray analysis as differentially regulated were subjected to validation by either qPCR using β2-microglobulin as a reference gene) or by ELISA (for the quantification of protein secretion). Figure 5a shows that T_MOG_ stimulation with MOG35-55 resulted in increased IL-1β protein secretion and that co-incubation with CBD lowers it back to control level. Moreover, T_MOG_ stimulation with MOG35-55 increased IL-3 secretion by *circa* tenfold and this level was suppressed by CBD by 40 % (Fig. 5b).

Table 6 shows examples of qPCR analysis of selected gene products in stimulated T_MOG_ cells. T_MOG_ stimulation with MOG35-55 resulted in a high upregulation of the mRNA level of *Xcl1* chemokine (62.7-fold) and this level was suppressed by CBD by *circa* fourfold. The expression of *Il12a* was also increased by MOG35-55 (4.3-fold) and reduced to 2.6-fold when CBD was added to MOG35-55-stimulated T_MOG_ cells. Stimulation of T_MOG_ cells with MOG35-55 increased also the expression of *Dusp6* by 2.4-fold that was further increased by co-incubation with CBD (by around 40 %). The table also shows several other genes which transcription was significantly enhanced by CBD alone including *Btla, Lag3*, and *Irf4*. The transcription of *Btla, Lag3*, and of *Irf4* was further increased when MOG35-55 and CBD were added together. Interestingly, *Il10* mRNA was increased only when MOG35-55 and CBD were applied together but not following MOG35-55 or CBD given alone. These observations are in agreement with the mRNA expression observed using the gene profiling method.

**Table 6:** qPCR validation of various transcripts in mRNA prepared from MOG35-55-stimulated T_MOG_ cells and the effect of CBD

Symbol	Gene name	MOG alone [fold] ^$^	CBD+MOG [fold] ^$^	CBD alone [fold] ^$^	One-way ANOVA
*Xcl1*	Chemokine (C Motif) Ligand 1	62.7 ± 0.7^##^	17.9 ± 11.4**	1.6 ± 0.1	F(3,8) = 12.6, *p* < 0.01
*IL12a*	Interleukin 12	4.3 ± 0.1^#^	2.6 ± 0.3*	0.8 ± 0.1	F(3,8) = 6.0, *p* < 0.05
*Btla*	B- and T-lymphocyte attenuator	2.8 ± 0.4^##^	7.1 ± 0.4***	6.0 ± 0.5^###^	F(3,8) = 60.9, *p* < 0.001
*Dusp6*	Dual specificity phosphatase 6	2.4 ± 0.4	3.3 ± 0.7^#^	1.9 ± 0.3	F(3,8) = 3.3, p = 0.07
*Irf4*	Interferon regulatory factor 4	3.4 ± 0.6^##^	7.9 ± 0.5***	3.7 ± 0.3^##^	F(3,8) = 43.3, *p* < 0.001
*Lag3* ^&^	Lymphocyte-activation gene 3	11.5 ± 0.3^###^	25.9 ± 2.3***	5.8 ± 1.5*	F(3,8) = 114, *p* < 0.001
*IL10* ^&^	Interleukin 10	1.5 ± 0.3	3.4 ± 0.8^#^	1.4 ± 0.3	F(3,11) = 3.6, *p* = 0.05

^$^Data are expressed as fold change vs control, not stimulated cells (equal to 1). ^#^ *p* < 0.05, ^##^ *p* < 0.01, ^###^ *p* < 0.01 *vs* control T_MOG_ cells. *p < 0.05, **p < 0.01, ***p < 0.001 *vs* MOG-35-55-stimulated T_MOG_ cells. ^&^results reported previously [25]

### Weighted gene co-expression network analysis (WGCNA)

**Fig. 6:**
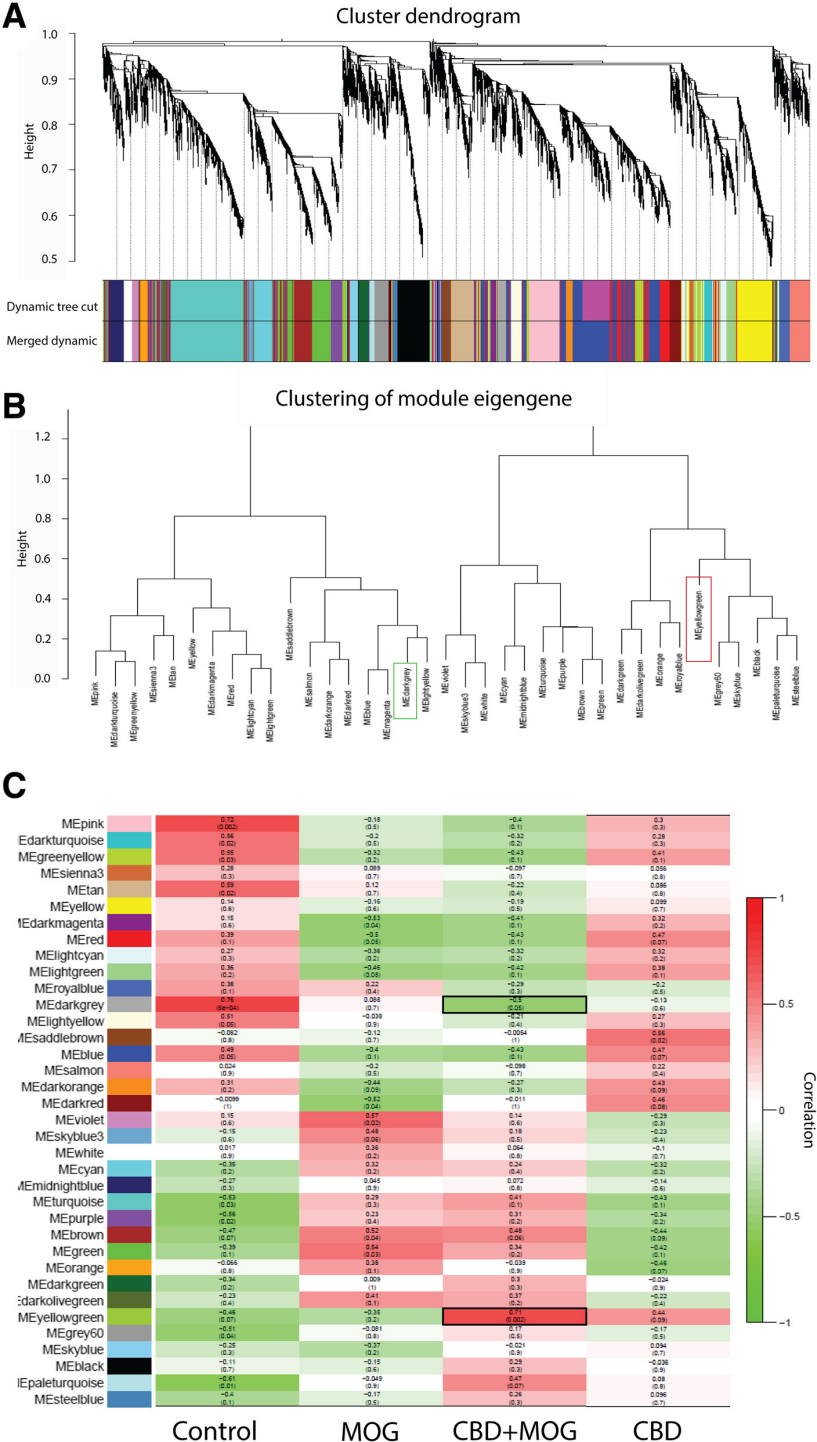
WGCNA-clustered gene modules and their correlation to CBD effect in MOG35-55-stimulated T_MOG_ cells. WGCNA analysis identifies distinct modules of coexpressed genes. **a** Dendrogram showing topological overlap of genes and their relation to modules which are *color-coded* below. **b** clustering of resulting modules. *Color* frames indicate the main modules that are positively (*red frame*) and negatively (*green frame*) correlated to CBD effect in MOG35-55-stimulated T_MOG_. **c** module-trait relationship plots showing Spearman’s correlation between module principal components (a.k.a. module eigengenes [ME]; in *color-coded rows*) and the treatments. The *top value* in each square represents the correlation coefficient between the ME and the treatment. The *bottom value* in parentheses is the relevant *p* value. Modules highly correlated with CBD+MOG35-55 treatment are emphasized using *black frames*

We applied WGCNA analysis and clustering to complement the functional characterization of the changes in gene expression induced by CBD in activated T_MOG_. As mentioned in Methods, WGCNA clusters functionally related transcripts into modules in an unsupervised manner based on the expression pattern across the samples. The result of this clustering can be viewed as a dendrogram (Fig. 6a) in which each branch corresponds to a group of co-expressed genes (a module) that is designated a color and will be referred to by its color in the rest of the manuscript.

WGCNA analysis grouped the affected transcripts into 36 discrete modules (Fig. 6b). Pearson correlation of these modules revealed modules that were the most significantly correlated to each of the treatments (Fig. 6c). The *darkgrey* module was shown to be the most negatively correlated (*r* = −0.5, *p* = 0.05) and the *yellowgreen* as the most positively correlated (*r* = 0.71, *p* = 0.002) to the combined CBD+MOG35-55 treatment. This stays in contrast to MOG35-55 effect where the *darkgrey* gene coexpression module was recognized as positively correlated (*r* = 0.088, *p* = 0.7) and the *yellowgreen* module negatively correlated (*r* = 0.35, *p* = 0.2). This further indicates that CBD reverses gene interactions’ patterns induced by MOG35-55 stimulation of T_MOG_. In search for functional interpretation of these associations, we annotated the CBD+MOG35-55-correlated modules with GO categories to highlight the main biological functions linked to the CBD effect in stimulated T_MOG_ cells. The GO categories identified within the *darkgrey* module (containing 94 genes Additional file 1: Table S2) seemed to be associated predominantly with “*antigen processing and presentation of exogenous peptide antigen via MHC class II*” (GO:0019882, GO:0002504 GO:0019886 GO:0002495 GO:0002478 GO:0019884). This is probably due to the high representation within this module of genes regulating antigen processing, *e.g.*, H2-Ab1, CD9, Tmem66, CD74, Tlr2, and Fcrla. Other GO categories identified within this the *darkgrey* module include “*immune system processes*” (GO:0002376), “*defense response*” (GO:0006952), “*inflammatory response*” (GO:0006954), “*reactive nitrogen species metabolic process*” (GO:2001057), “*regulation of chemokine production*” (GO:0032642, all within Biological Process terms), and “*binding*” (GO:0005488; within Molecular Function terms).

The *yellowgreen* module (containing 48 genes Additional file 1: Table S2) includes genes such as Ptpnm2, Slc30a1 and Btla. GO analysis qualified the *yellowgreen* assigned terms into “*regulation of immune system processes*” (GO:0002682), “*response to stimulus*” (GO:0048583), “*signaling*” (GO:0023051), “*cell communication*” (GO:0010646), “*cell adhesion*” (GO:0030155), “*signal transduction*” (GO:0009966; within Biological Process terms), “*guanyl-nucleotide exchange factor activity*” (GO:0005085; within Molecular Function terms), and “*external side of plasma membrane*” (GO:0009897; Cellular Component terms). In summary, the WGCNA analysis demonstrated that the main effect of CBD is to restrain the MOG35-55 activation of T_MOG_ predominantly by targeting processes regulating antigen processing and presentation.

## Discussion

In the present study, we characterized the CBD effects on the transcriptional activity of mouse MOG35-55-activated encephalitogenic T_MOG_ cells, the latter known to induce autoimmune pathologies. We previously showed that CBD decreases IL-17 and IL-6 mRNA production and protein secretion in MOG35-55-stimulated T_MOG_ co-cultured with APC while increasing anti-inflammatory IL-10 mRNA synthesis [24, 26]. In parallel, we showed that CBD negatively regulates STAT3 phosphorylation while enhancing STAT5 activity, main positive and negative regulators of Th17 differentiation, respectively [36], further confirming the regulatory effect of CBD on Th17 function of memory T cells [26]. The present gene profiling reveals that CBD targets a relatively small number of genes (163) within a large group of those significantly affected by MOG35-55 stimulation (reaching 1936 transcripts). Bioinformatics analysis performed on these CBD affected transcripts assembled them into various pathways and gene networks mediating the effects of CBD on the MOG35-55-activated memory T cells. In line with our previous studies, this gene profiling confirms that CBD affects various processes controlling “*Th17 differentiation*”, including “*IL-6 signaling*” (a pathway promoting memory Th17 generation) and anti-inflammatory “*IL10 signaling*” (known to restrain Th17 function and Th17-mediated autoimmunity [25, 37]).

CBD upregulated the transcription of 81 genes and downregulated 82 genes in MOG35-55-stimulated T_MOG_. For example, CBD was found to decrease the MOG35-55-upregulated mRNA levels of various proinflammatory mediators including interleukins (*Il3*, *Il1b*, and *Il12a*), chemokines (*Xcl1* and *Cxcl9*) and receptors (*Cxcr1*, *Ifngr1*, *Gpr83*), molecules known to be involved in Th17 generation and function [38–40]. In addition, CBD downregulated elements of various signaling cascades, including members of the TNF superfamily (e.g., *Tnfsf11*, *Tnfrsf18*), molecules mediating cell adhesion (e.g., *Amica1*) growth, structure, and metabolism (e.g., *Cdk5r1*, *Tubb2d*, *Dgat1*). The effect of CBD on the TNF superfamily members was found to be very diverse. This is not surprising since the TNF superfamily consists of a large number of molecules involved in multiple cellular processes including inflammation, cell growth, cell cycle, differentiation, metabolism, and cytokine production [41]. The mRNA of the cAMP-responsive element modulator (CREM) transcription factor was also significantly suppressed by CBD in activated encephalitogenic T_MOG_ cells. CREM activity was shown to control IL-2 and IL-17 expression during CD4 lineage commitment and distribution [42]. Indeed, human autoimmune T cells were shown to display increased expression of CREM which accounted for their increased IL-17 release and autoimmunity (*e.g.*, in systemic lupus erythematosus and MS-like pathologies [43, 44]). Thus, the decrease in *Crem* mRNA found here may serve as an important contribution to the CBD-induced suppression of the Th17-like activity of the T_MOG_ cells. This downregulation of *Crem* mRNA by CBD does not seem to be mediated *via* the cannabinoid receptors CB1 and CB2, both known to affect adenylyl cyclase activity and cAMP formation [45, 46]. This is due to the fact that CBD was shown to have negligible affinity toward these receptors [47]. Moreover, we showed previously that CBD effects on T_MOG_ do not involve these receptors [12, 24].

Several of the MOG35-55-induced transcripts were preserved at high levels or even further upregulated in the presence of CBD. *Egr2* transcription factor was one of the highest upregulated representative of this group along with a number of cytokines (*Csf2*, *Ifng*, *Tnf*), chemokines (*Ccl3*, *Ccl4*, *Cxcl10*) and regulatory glycoproteins (*Sema7a*). This gene expression pattern may indicate a promotion by CBD of tolerogenic conditions driving exhaustion of T cells (anergy) and thus leading to the functional deactivation of T cells [48]. Indeed, EGR2 is an acknowledged T cell anergy marker which induction turns on a genetic program driving cell cycle arrest, eventually dampening immune responses [49, 50] including IL-17 production [51, 52]. Indeed, we have recently shown that CBD induces Egr2-dependent anergy in encephalitogenic T cells and that CBD enhances the expression of several other anergy signature genes including *Il10* and *Lag3* [26].

MOG35-55-upregulated *Ifng mRNA* production and secretion were not affected by CBD in stimulated T_MOG_ cells [24]. This result was reproduced by the current microarray analysis showing no effect of CBD on the IFNγ upstream transcription factor *Tbx21* mRNA. On the other hand, the present results show that CBD has a potent enhancing effect on several IFN-dependent transcripts including *Irf4*, *Ifit3*, *Rsad2*/*Viperin*, and *Mx2*. Type I (IFN-α and -β) and type II (IFN-γ) interferons are known to mediate innate immune responses and inhibit the proliferation of virus-infected cells [53]. IFNs exert antiproliferative and immunomodulatory effects and IFNγ expressing cells were shown to counteract Th17 activity [54]. Alterations in IFN-stimulated gene patterns (including in *Ifit*, *Mx2*, *Cxcl10*) and/or in Jak/STAT activity, a main IFN-dependent pathway, cause a failure in immunological tolerance resulting in T cell-mediated autoimmune pathologies (*e.g.*, type I diabetes) [55]. Thus, we assume that CBD-enhanced IFN-controlled pathways may contribute to CBD anti-proliferative effects in activated memory T cells, as already observed by us previously [12].

Interferon regulatory factor 4 (IRF4, a lymphocyte-specific nuclear factor) was shown to be a core promoter of other IFN-dependent genes such as *Rsad2* and *Ifit3* [56]. IRF4 was reported to mediate the differentiation of naive CD4+ T cells into various T helper lineages [57]*.* IRF4 potently synergizes with NFAT signaling to enhance the promoter activity of anti-inflammatory IL-4 cytokine [58]. Indeed, in our hands *Nfatc1* mRNA, an element of the NFAT signaling axis, was upregulated by CBD.

The transcription of several genes was downregulated by MOG35-55 stimulation. This group of genes included IL-17 receptors (*Il17re* variant 1 and 2, *Il17rc*). Interestingly, CBD did not reverse this *Il17r* mRNA downregulation suggesting that CBD does not interfere with intrinsic negative feedback mechanisms restraining the activity of memory T cells.

Antigen-induced T cell activation occurs following peptide presentation by major histocompatibility complex class II (MHCII) molecules on APC cells. A number of molecules negatively regulate these APC/T interactions [59]. Our study shows that CBD is a powerful inducer of negative regulators of APC/T interactions as CBD increased the mRNA levels of: B and T lymphocyte attenuator (*Btla*/*Cd272*); lymphocyte-activation gene 3 (*Lag3/Cd223*); cluster of differentiation 69 (*Cd69*) and of T cell receptor associated transmembrane adaptor 1/T-cell receptor interacting molecule (*Trat1*/TRIM). These molecules are members of a broad, structurally related group of immunoglobulin receptors that were found to serve as markers of various inducible T cell regulatory phenotypes involved in Th17 suppression and amelioration of MS-like pathologies [59–62]. For example, LAG3 is a CD4 receptor homologue that by interfering with MHCII on APC upon antigen exposure inhibits the function and expansion of memory T cells [63] decreasing IL-17 and increasing IL-10 secretions, this way preventing autoimmunity in mice [64, 65]. Mice lacking the CD69 transmembrane receptor develop aggravated forms of autoimmune pathologies including arthritis, contact dermatitis, allergic asthma, and autoimmune myocarditis [66]. Indeed, CD69-deficient T cells demonstrate increased differentiation into Th17 cells, higher IL-17 release as well as Jak3/Stat5 and RORγt pathways’ increased activities [67] (Martin 2010). Similarly, BTLA ligation with its ligand, herpes virus entry mediator (HVEM/TNFRSF14), was shown to attenuate T cell proliferation [68, 69] and to promote anergy [70]. Functional BTLA/HVEM pathway maintains immune tolerance and prevents autoimmunity (for review see [71] and BTLA-deficiency in mice leads to T cell-mediated rheumatoid arthritis, lymphocytic infiltration, autoimmune hepatitis-like diseases, and EAE [72–74]. CTLA-4 (CD152) is an inhibitory receptor which in its inactive state is located intracellularly and upon demand is trafficked by TRAT1/TRIM protein (that is upregulated by CBD) to the membrane [75]. CTLA-4 negatively competes on T cells with CD28, a co-stimulatory T cell molecule, in binding to the co-activators CD80 and CD86 on APC cells [76, 77]. TRAT1/TRIM-dependent increase in CTLA4 expression led to suppression of encephalitogenic T cells expansion and of EAE development [78]. In agreement with this, impaired CTLA-4 expression on T cells was found to correlate with increased IL-17 release and more severe EAE [79]. Moreover, CTLA-4-deficient mice show an autoimmune phenotype with T cell-mediated organ destruction [80–82]*.* Thus, CBD upregulation of these powerful inhibitors of APC/T communication should exert robust immunoregulatory effect in MOG35-55 activated T_MOG_ resulting in their suppressed activity. Indeed, complement WGCNA analysis confirmed the inhibitory role of CBD in processes related to antigen presentation and processing and APC/T_MOG_ interactions. Moreover, these observations are in agreement with our previous report showing that CBD decreases the activation of B cells (the main APC in the periphery) incubated with T_MOG_ as well as MHCII expression on these cells [26].

Reactive oxygen species generated primarily by macrophages and damaged neurons, increase immune response and T cell activation, propagate demyelination and neurodegeneration [83]. The present gene profiling links the CBD effects to nuclear factor-erythroid 2-related factor 2 (Nfe2l2/Nrf2)-driven antioxidant genetic program, known to exert immunoregulatory effects [84, 85]. Our results show that CBD enhances the expression of Nrf2 target genes including Heme oxygenase-1 (Hmox-1 encoding HO-1 protein) and metallothioneins (*Mt1* and *Mt2*). Indeed, Mt(−/−) mice show increased macrophage and T-lymphocyte infiltration into the CNS as well as increased cytokine release, oxidative stress and axon damage as compared to their wildtype EAE littermates [86]. HO-1 was shown to diminish inflammatory reactions, *e.g.*, *via* induction of the anti-inflammatory IL10 cytokine and modulation of Jak/STAT pathway [87]. Indeed, pharmacological or genetic induction of *Nrf2*-dependent anti-oxidant genes (i.e., *Hmox1*, *Mt1*, and *Mt2*) in EAE mice led to reduction in IL-17 levels and decreased EAE severity [88]. Moreover, Hmox1−/− C57BL/6 mice develop enhanced EAE-like paralysis and CNS demyelination while HO-1 restoration diminished EAE progression and relapses [89]. Interestingly, this protective effect of HO-1 has been associated with inhibition of MHCII expression by APCs via mechanisms involving carbon monoxide, an antioxidant product of HO-1 activity. Thus, CBD induction of Nfe2l2/Nrf2-dependent antioxidant genes may be one of the key mechanisms driving the anti-inflammatory effects of this cannabinoid in T_MOG_ cells and EAE. Similar CBD regulation of anti-oxidant genes was reported by us in quiescent and LPS-stimulated microglial cells [30, 90]. These findings suggest that Nrf2-dependent immunoregulation may be an important feature of this cannabinoid contributing to its anti-inflammatory effects. However, we cannot rule out that CBD is also affecting several other pathways, e.g., receptors for A2A as well as dimers of CB2/5HT1A [91]. In addition, CBD was shown to increase the endocannabinoid levels, known to modulate immune functions [92], thus it may indirectly exert immunoregulatory actions on T cells through CB2 receptors, previously suggested to modulate T cell-mediated autoimmunity [93].

## Conclusions

In summary, gene expression profiling followed by bioinformatics analyses demonstrate that CBD exerts its immunoregulatory effects in activated memory T_MOG_ cells *via* several mechanisms: (a) by suppressing proinflammatory transcription and inhibiting pathways that favor Th17 differentiation and function, (b) by promoting a transcriptional set up leading to T cell exhaustion/tolerance, (c) by enhancing IFN-dependent anti-proliferative program, (d) by potentiating intrinsic T cell co-inhibitory mechanisms hampering APC/T communication and efficient antigen presentation, and (e) by inducing antioxidant milieu resolving inflammation.

## Abbreviations

*APC* antigen-presenting cells, *CBD* cannabidiol, *EAE* experimental autoimmune encephalomyelitis, *GO* gene ontology, *IFN* interferon, *IL* interleukin, *IPA* ingenuity pathway analysis, *MOG35-55* myelin oligodendrocyte glycoprotein 35-55, *T*_*MOG*_ MOG35-55-specific T cells, *WGCNA* weighted gene co-expression network analysis

## Additional files

Additional file 1: Table S1. List of primers used for qPCR validation of mRNA levels of selected genes in purified T_MOG_ cells. **Table S2.** WGCNA-assigned gene content of *yellowgreen* and *darkgrey* modules, classified as being the most correlated with CBD treatment in MOG35-55-stimulated T_MOG_. (DOC 233 kb)
