# Supplementary material for: Pathways and gene networks mediating the regulatory effects of cannabidiol, a nonpsychoactive cannabinoid, in autoimmune T cells
Source: J Neuroinflammation. 2016 Jun 3;13:136. doi: 10.1186/s12974-016-0603-x (PMC4891926; doi:10.1186/s12974-016-0603-x)
Supplement: Additional file 1: Table S1. — List of primers used for qPCR validation of mRNA levels of selected genes in purified TMOG cells. Table S2. WGCNA-assigned gene content of yellowgreen and darkgrey modules, classified as being the most correlated with CBD treatment in MOG35-55-stimulated TMOG. (DOC 233 kb) [file 12974_2016_603_MOESM1_ESM.doc]

**Table S1. List of primers used for qPCR validation of mRNA levels of selected genes in purified T_MOG_ cells.**

| **Gene** | **Accession Number** | **Forward** | **Reverse** |
| --- | --- | --- | --- |
| Il12a | NM_008351.2 | CTCCATCGCTTCTCTCATATTC | AGTTCTTGCTCTTCTGCTAAC |
| Xcl1 | NM_008510.1 | CACCAGCACAGCGATAAC | GCAATGGGTTTGGGAACT |
| Btla | NM_177584.3 | GTTAAGGGAAGGTCTCTCATTG | GCTGGGCATGAGCAAATA |
| Dusp6 | NM_026268.3 | GCAATACTTTGGGTTGGTTTC | GCAAGTTAATTCTCCCTTCCT |
| Irf4 | NM_013674.1 | AGTGCTTTGAGGAACATGAG | TGAAGATTGTGAAGCCTGTAG |
| Lag3 | NM_008479.2 | CTGCCTTAGAACATGGGATTC | CCATCTCCGTCTCCAGTT |
| Il10 | NM_010548.2 | CCTTTGCTATGGTGTCCTTTC | GGATCTCCCTGGTTTCTCTT |
| β_2_mg | NM_009735 | AGTTCCACCCGCCTCACATTGAAA | TCGGCCATACTGGCATGCTTAACT |

**Table S2. WGCNA-assigned gene content of *yellowgreen* and *darkgrey* modules, classified as being the most correlated with CBD treatment in MOG35-55-stimulated T_MOG_.**

| **Accession** | **Symbol** | **Definition** | **Correlation**  **to**  **eigengene** | **p value** |
| --- | --- | --- | --- | --- |
| **DARKGREY MODULE** | |  |  |  |
| NM_024444 | Cyp4f18 | Cytochrome P450, family 4, subfamily f, polypeptide 18 | 0.96 | 0.0092 |
| NM_010187.1 | Fcgr2b | Fc fragment of IgG, low affinity IIb, receptor (CD32) | 0.95 | 0.0247 |
| NM_008437.1 | Napsa | Napsin A aspartic peptidase | 0.94 | 0.0006 |
| NM_009304.1 | Syngr2 | Synaptogyrin 2 | 0.94 | 0.0037 |
| NM_011487.1 | Stat4 | Signal transducer and activator of transcription 4 | 0.93 | 0.0244 |
| NM_178911.2 | AI132321 | Phospholipase D family, member 4 | 0.92 | 0.0011 |
| NM_001012520.1 | Klri1 | Killer cell lectin-like receptor family I member 1 | 0.92 | 0.0014 |
| NM_009841.2 | Cd14 | Cluster of differentiation 14 | 0.91 | 0.0051 |
| NM_011136.1 | Pou2af1 | POU class 2 associating factor 1 | 0.91 | 0.0195 |
| NM_011905.2 | Tlr2 | Toll-like receptor 2 | 0.91 | 0.0065 |
| NM_008538 | Marcks | Myristoylated alanine-rich protein kinase C substrate | 0.91 | 0.0312 |
| NM_153513.1 | BC028528 | cDNA sequence BC028528 | 0.90 | 0.0313 |
| NM_019511.1 | Ramp3 | Receptor (calcitonin) activity modifying protein 3 | 0.90 | 0.0407 |
| NM_008535.1 | Lyl1 | Lymphoblastic leukemia associated hematopoiesis regulator 1 | 0.90 | 0.0090 |
| NM_207105.1 | H2-Ab1 | Histocompatibility 2, class II antigen A, beta 1 | 0.90 | 0.0024 |
| NM_016851.1 | Irf6 | Interferon Regulatory Factor 6 | 0.90 | 0.0128 |
| NM_019511.1 | Ramp3 | Receptor (calcitonin) activity modifying protein 3 | 0.89 | 0.0063 |
| NM_023065.2 | Ifi30 | Interferon, Gamma-Inducible Protein 30 | 0.89 | 0.0022 |
| NM_007779.1 | Csf1r | Colony Stimulating Factor 1 Receptor | 0.89 | 0.0062 |
| NM_007792.2 | Csrp2 | Cysteine and glycine-rich protein 2 | 0.89 | 0.0294 |
| NM_146184.2 | BC025206 | UDP-GlcNAc:betaGal beta-1,3-N-acetylglucosaminyltransferase 8 | 0.89 | 0.0101 |
| NM_020001.1 | Clec4n | C-type lectin domain family 4, member n (B3gnt8) | 0.89 | 0.0032 |
| NM_025598.1 | 2700038C09Rik | RIKEN cDNA 2700038C09 gene | 0.89 | 0.0011 |
| XM_128971.2 | Klhl14 | Kelch-like family member 14 | 0.89 | 0.0027 |
| NM_019511.1 | Ramp3 | Receptor (calcitonin) activity modifying protein 3 | 0.89 | 0.0596 |
| NM_145141.1 | Fcrla | Fc receptor-like A | 0.89 | 0.0352 |
| NM_011093.1 | Pira6 | Paired-Ig-like receptor A6, transcript variant 2. | 0.89 | 0.0006 |
| NM_013642.1 | Dusp1 | Dual specificity phosphatase 1 | 0.89 | 0.0108 |
| NM_010858.3 | Myl4 | Myosin, light polypeptide 4 | 0.88 | 0.1481 |
| NM_172742 | Mtmr10 | Myotubularin related protein 10 | 0.88 | 0.0942 |
| NM_178382.2 | Flrt3 | Fibronectin leucine rich transmembrane protein 3 | 0.88 | 0.0228 |
| NM_009976.2 | Cst3 | Cystatin C | 0.88 | 0.0001 |
| NM_009270.2 | Sqle | Squalene Epoxidase | 0.88 | 0.0027 |
| NM_009892 | Chi3l3 | Chil3 chitinase-like 3 | 0.88 | 0.0125 |
| NM_177171.3 | Heatr5a | HEAT repeat containing 5A | 0.88 | 0.0007 |
| NM_009304.1 | Syngr2 | Synaptogyrin 2 | 0.87 | 0.0082 |
| NM_009892.1 | Chi3l3 | Chitinase 3-like 3 | 0.87 | 0.0015 |
| NM_174851.2 | Il28ra | Interleukin 28 receptor alpha | 0.87 | 0.0370 |
| NM_026388.2 | 1200015A19Rik | RIKEN cDNA 1200015A19 gene. | 0.87 | 0.0006 |
| NM_019948.1 | Clecsf9 | C-Type lectin domain family 4, member E | 0.87 | 0.0387 |
| NM_017370.1 | Hp | Haptoglobin | 0.87 | 0.0008 |
| NM_009304.1 | Syngr2 | Synaptogyrin 2 | 0.86 | 0.0131 |
| NM_009514.2 | Vpreb3 | Pre-B lymphocyte gene 3 | 0.86 | 0.0562 |
| NM_174851.2 | Il28ra | Interleukin 28 receptor alpha | 0.86 | 0.0026 |
| NM_133888.1 | Smpdl3b | Sphingomyelin phosphodiesterase, acid-like 3B | 0.86 | 0.0538 |
| NM_011026.1 | P2rx4 | Purinergic receptor P2X, ligand gated ion channel, 4 | 0.86 | 0.0180 |
| NM_026301.1 | Rnf125 | Ring finger protein 125 | 0.85 | 0.0002 |
| NM_007657.2 | Cd9 | CD9 antigen | 0.85 | 0.0238 |
| NM_007895.2 | Ear2 | Eosinophil-associated, ribonuclease A family, member 2 | 0.85 | 0.0704 |
| NM_023716.1 | Tubb2b | Tubulin, beta 2b | 0.85 | 0.1325 |
| NM_013710.1 | Fgd2 | FYVE, RhoGEF and PH domain containing 2 | 0.85 | 0.0162 |
| NM_010511.1 | Ifngr1 | Interferon Gamma Receptor 1 | 0.85 | 0.0035 |
| NM_133885 | Osbpl9 | Oxysterol binding protein-like 9 | 0.84 | 0.0577 |
| NM_028696.2 | Obfc2a | Oligonucleotide/oligosaccharide-binding fold containing 2A | 0.84 | 0.0000 |
| NM_001017422.1 | Ear4 | Eosinophil-associated, ribonuclease A family, member 4 | 0.84 | 0.0554 |
| NM_013739.1 | Dok3 | Docking protein 3 | 0.84 | 0.0720 |
| NM_134099.1 | Fbxo4 | F-box protein 4 | 0.84 | 0.0169 |
| NM_019706.2 | Rnf138 | Ring finger protein 138, transcript variant 2 | 0.84 | 0.0004 |
| NM_026428.1 | Dcxr | Dicarbonyl L-xylulose reductase | 0.84 | 0.0289 |
| NM_153059.1 | Tmem5 | Transmembrane protein 5 | 0.83 | 0.0038 |
| NM_011182.2 | Pscd3 | Pleckstrin homology, Sec7 and coiled-coil domains 3 | 0.83 | 0.0516 |
| NM_008039.1 | Fpr-rs2 | Formyl peptide receptor 2 | 0.83 | 0.1148 |
| NM_010545.2 | CD74 | Invariant polypeptide of major histocompatibility complex, class II antigen-associated (Cd74), transcript variant 2 | 0.83 | 0.0021 |
| XM_195168.3 | Pla2g4f | Phospholipase A2, group IVF | 0.83 | 0.0394 |
| NM_144923.2 | Blvrb | Biliverdin reductase B (flavin reductase (NADPH)) | 0.83 | 0.0082 |
| NM_026405.2 | Rab32 | RAB32, member RAS oncogene family | 0.82 | 0.0262 |
| NM_133977.1 | Trf | Transferrin | 0.82 | 0.0395 |
| NM_009970.1 | Csf2ra | Colony stimulating factor 2 receptor, alpha, low-affinity (granulocyte-macrophage) | 0.82 | 0.0491 |
| NM_025436.1 | Sc4mol | Sterol-C4-methyl oxidase-like. | 0.81 | 0.1031 |
| NM_182994.1 | Arl5a | ADP-ribosylation factor-like 5A | 0.81 | 0.0078 |
| NM_011090.1 | Pira3 | Paired-Ig-like receptor A3 | 0.81 | 0.0056 |
| NM_010070.3 | Dok1 | Docking protein 1 | 0.80 | 0.0000 |
| NM_019453.1 | Mefv | Mediterranean fever | 0.80 | 0.0174 |
| NM_008026 | Fli1 | Fli-1 Proto-Oncogene, ETS Transcription Factor | 0.80 | 0.0001 |
| NM_011216.1 | Ptpro | Protein Tyrosine Phosphatase, Receptor Type, O | 0.79 | 0.0001 |
| NM_023716.1 | Tubb2b | Tubulin, beta 2b | 0.79 | 0.0909 |
| NM_026432.2 | Tmem66 | Transmembrane protein 66 | 0.78 | 0.0003 |
| NM_008853.2 | Pja1 | Praja1, RING-H2 motif containing | 0.78 | 0.0728 |
| NM_144906 | Sgip1 | SH3-domain GRB2-like (endophilin) interacting protein 1 | 0.76 | 0.0917 |
| NM_016796.2 | Vamp4 | Vesicle-associated membrane protein 4 | 0.76 | 0.0010 |
| NM_134037.2 | Acly | ATP citrate lyase | 0.75 | 0.0000 |
| NM_028347.1 | Neil1 | Nei endonuclease VIII-like 1 (E. coli) | 0.75 | 0.0923 |
| NM_053112.1 | Ear10 | Eosinophil-associated, ribonuclease A family, member 10 | 0.73 | 0.1894 |
| NM_012021 | Prdx5 | Peroxiredoxin 5 | 0.73 | 0.0637 |
| NM_022019.2 | Dusp10 | Dual specificity phosphatase 10 | 0.72 | 0.1337 |
| NM_017372 | Lyzs | Lysozyme 2 | 0.69 | 0.0717 |
| NM_028784.2 | F13a1 | Coagulation factor XIII, A1 subunit | 0.68 | 0.1113 |
| NM_010046.2 | Dgat1 | Diacylglycerol O-acyltransferase 1 | 0.64 | 0.1821 |
| NM_013653.1 | Ccl5 | Chemokine (C-C motif) ligand 5 | 0.63 | 0.3719 |
| NM_025623.1 | Nipsnap3b | Nipsnap homolog 3A (C. elegans) | 0.61 | 0.2042 |
| NM_007984.1 | Fscn1 | Fascin actin-bundling protein 1 | 0.59 | 0.1814 |
| NM_027878.1 | 1200002N14Rik | RIKEN cDNA 1200002N14 gene | 0.57 | 0.6722 |
| NM_025289.1 | Tbrg1 | Transforming growth factor beta regulated gene 1 | 0.55 | 0.2941 |
| **YELLOWGREEN MODULE** | |  |  |  |
| NM_011256.1 | Pitpnm2 | Phosphatidylinositol transfer protein, membrane-associated 2 | 0.96 | 0.7174 |
| NM_145506.2 | Epb4.1l5 | Erythrocyte protein band 4.1-like 5 | 0.96 | 0.3783 |
| NM_009579 | Slc30a1 | Solute carrier family 30 (zinc transporter), member 1 | 0.91 | 0.4945 |
| NM_029857.2 | Tmco4 | Transmembrane and coiled-coil domains 4 | 0.92 | 0.6126 |
| NM_144953.1 | 1700019D03Rik | RIKEN cDNA 1700019D03 gene | 0.93 | 0.7023 |
| NM_007584.1 | Ddr1 | Discoidin domain receptor family, member 1 | 0.92 | 0.8399 |
| NM_010685.2 | Lamp2 | Lysosomal-associated membrane protein 2 | 0.91 | 0.9658 |
| NM_173760 | Ppip5k2 | Diphosphoinositol pentakisphosphate kinase 2 | 0.92 | 0.4166 |
| NM_172664.2 | Tlk1 | Tousled-like kinase 1 | 0.91 | 0.3864 |
| NM_027871.1 | Arhgef3 | Rho guanine nucleotide exchange factor (GEF) 3 | 0.90 | 0.3373 |
| NM_177628.3 | BC065085 | cDNA sequence BC065085 | 0.90 | 0.9915 |
| XM_109657.3 | Fnip1 | Folliculin interacting protein 1 | 0.85 | 0.3700 |
| NM_178653.2 | Sccpdh | Saccharopine dehydrogenase (putative) | 0.84 | 0.2121 |
| NM_198297 | Trat1 | T cell receptor associated transmembrane adaptor 1 | 0.85 | 0.2862 |
| NM_146091.2 | Atl3 | Atlastin GTPase 3 | 0.84 | 0.8740 |
| NM_177584.3 | Btla | B and T lymphocyte associated | 0.85 | 0.6944 |
| NM_172727.1 | Fam221a | Family with sequence similarity 221, member A | 0.81 | 0.3279 |
| NM_001017959.1 | Lamp2 | Lysosomal-associated membrane protein 2 | 0.85 | 0.6277 |
| NM_178917.2 | Arrdc3 | Arrestin domain containing 3 | 0.86 | 0.0235 |
| NM_027342.1 | Fam162a | Family with sequence similarity 162, member A | 0.82 | 0.4201 |
| NM_175098.2 | Ccdc126 | Coiled-coil domain containing 126 | 0.82 | 0.2707 |
| NM_010278.1 | Gfi1 | Growth factor independent 1 | 0.80 | 0.1343 |
| NM_133218.1 | Gig1 | Zinc finger protein 704 (Zfp704), | 0.82 | 0.5376 |
| NM_146139.1 | Vav3 | Vav 3 oncogene | 0.77 | 0.8300 |
| NM_016846.2 | Rgl1 | Ral guanine nucleotide dissociation stimulator,-like 1 | 0.84 | 0.0722 |
| NM_173753.3 | Fnip1 | Folliculin interacting protein 1 | 0.79 | 0.3452 |
| NM_017480.1 | Icos | Inducible T-cell co-stimulator | 0.82 | 0.5134 |
| NM_008397 | Itga6 | Integrin alpha 6 | 0.81 | 0.1393 |
| NM_144875.1 | Rab7l1 | RAB7, member RAS oncogene family-like 1 | 0.79 | 0.8459 |
| NM_207225.1 | Hdac4 | Histone deacetylase 4 | 0.78 | 0.9606 |
| NM_023680 | Tnfrsf22 | Tumor necrosis factor receptor superfamily, member 22 | 0.80 | 0.3926 |
| NM_001024617.2 | Inpp4b | Inositol polyphosphate-4-phosphatase, type II | 0.80 | 0.6993 |
| NM_053100.1 | Trim8 | Tripartite motif protein 8 | 0.77 | 0.7416 |
| NM_011930.2 | Clcn7 | Chloride channel 7 | 0.80 | 0.2722 |
| NM_172952.1 | Gphn | Gephyrin | 0.74 | 0.7391 |
| NM_001025597.1 | Ikzf1 | IKAROS family zinc finger 1 transcript variant 1 | 0.77 | 0.3689 |
| XM_144142.3 | Klhl21 | Kelch-like 21 | 0.73 | 0.0462 |
| NM_021465 | Stag2 | Stromal antigen 2 | 0.76 | 0.3079 |
| NM_013545.1 | Hcph | Protein tyrosine phosphatase, non-receptor type 6 | 0.72 | 0.0999 |
| NM_028314.1 | 2700097O09Rik |  | 0.69 | 0.5087 |
| NM_026988.1 | Ptms | Parathymosin | 0.67 | 0.6323 |
| NM_011756.3 | Zfp36 | Zinc finger protein 36 | 0.70 | 0.4380 |
| NM_025920 | Thap4 | THAP domain containing 4 | 0.67 | 0.7090 |
| NM_173734.2 | Tmem87a | Transmembrane protein 87A | 0.68 | 0.7684 |
| NM_009943.1 | Cox6a2 | Cytochrome c oxidase subunit VIa polypeptide 2 | 0.66 | 0.2684 |
| NM_029857.2 | Tmco4 | Transmembrane and coiled-coil domains 4 | 0.63 | 0.4095 |
| NM_001007465.1 | Rffl | Ring finger and FYVE like domain containing protein, variant 1 | 0.57 | 0.7734 |
| NM_153561.1 | Nudt6 | Nudix (nucleoside diphosphate linked moiety X)-type motif 6 | 0.65 | 0.4597 |
